# Books and Pamphlets Received

**Published:** 1887-12

**Authors:** 


### BOOKS AND PAMPHLETS RECEIVED.

*A System of Gynecology by American Authors.* Edited by Mathew D. Mann, A.M., M.D., Professor of Obstetrics and Gynecology in the Medical Department of the University of Buffalo, New York. Vol. I. illustrated with three colored plates and two hundred and one engravings on wood. Philadelphia: Lea Brothers & Co. 1887.

We have here a volume of 789 large octavo pages, neatly printed substantially bound in calf, and beautifully illustrated. The work is to appear in two large volumes, of which this is the first It is made up of papers contributed by specialist or eminent representatives in the particular line treated—a method which commends itself to the student and practitioner as calculated to bring out the ablest efforts of able men, stimulated by their enthusiasm in the subject and by that laudable pride and emulation which may always be expected by men who have been chosen by reason of their acknowledged ability and reputation in any particular department. The following are the special subjects treated in this volume, each of which evinces marked ability on the part of the several contributors:

"Historical Sketches of American Gynecology."

"Development of the Female Genitals." Henry I. Carrigues, A.M., M.D.

"The Anatomy of the Female Pelvic Organs." Henry C. Coe, M.D., M.R. C. S.

"Malformation of the Female Genitals." By Henry J. Carrigues, A.M., M.D.

"Gynecological Diagnosis." By Egbert H. Grandin, A.B., M.D.

"General Consideration of Gynecological Surgery." By E. C. Dudley, A. B., M. D.

"General Therapeutics." By Alexander J. Skene, M.D.

"Electricity in Gynecology." By Alphonso D. Rockwell, A.M., M.D.

"Sterility." By A. Reeves Jackson, A.M., M.D.

"Diseases of the Vulva." By Mathew D. Mann. A M., M.D.

"The Inflammatory Affections of the Uterus." C. D. Palmer, M.D.

"Subinvolution of the Vagina and Uterus." Thaddeus A. Reamy, A.M., M.D.

"Periuterine Inflammation." By Richard B. Maury, M.D.

"Pelvic Hæmatocele and Hæmatoma." By Ely Van de Walker, M.D.

We have not space for comment upon these papers, and will only add that we regard this among the ablest and most instructive works yet issued in the department of Gynecology.

*The Presbyterian Quarterly.*—We have before us the October number of the *Presbyterian Quarterly Review*, being the second number of this new Quarterly published in Atlanta, Georgia, under the control of an association composed of a number of able and eminent divines in the Southern States. The immediate editors are: Rev. G. B. Strickler, D.D.; Rev. E. H. Barnett, D.D. The managing editor is the Rev. Geo. Summey, of Chester, S. C., to whom communications, exchanges, books, MS, etc., should be addressed. Each copy contains 80 pages of reading matter.

The *Quarterly* will represent the views and literature of the Presbyterian Church in the United States. A large number of articles on timely and important subjects has been engaged, and the character and learning of those who conduct it are a guarantee of the high standard and excellence to which this journal aims to attain. The specimen before us contains the following able and interesting papers:

"Spurious Religious Excitements." By R. L. Dabney, D.D., LL.D.

"Denominational Colleges." By W. M. Grier, D.D.

"Restoration of the Jews." By A. W. Mell, D.D., LL.D.

"Nineteenth Century Evangelism." By T. D. Witherspoon, D.D., LL.D.

"Organic Union." By C. R. Vaughn, D.D.

"The McGlynn Affair" By John McLawrin.

"The Hebrew Movement—Its Past, Present and Future."

"The Pseudo-Scientific View of Miracles." By R. R. Howison.

"Reasons for Reunion." J. M. Otts, D.D.

"The Moral Character of George Elliot." By Jas. H. Smith.

"Criticisms and Reviews." By Drs J. L. Girardeau, H. C. Alexander, B. B. Warfield, C. R. Hemphill, Geo. D. Armstrong, and Rev. R. A. Webb.

Terms, $3 00 per annum.

*Treatise on Human Physiology* for the use of Students and Practitioners of Medicine, by Henry C. Chapman, M. D., Professor of Institutes of Medicine and Medical Jurisprudence in the Jefferson Medical College of Philadelphia; Member of the College of Physicians of Philadelphia; of the Academy of National Sciences of Philadelphia; of the American Philosophical Society and of the Zoological Society of Philadelphia. Philadelphia: Lea Brothers & Co. 1887.

The above is a work of 945 octavo pages, well bound in calf, neatly printed in plain type and containing numerous illustrations. It is very recent, being just issued from the press, and is well up with the latest advances in physiological science. The general plan of the work and the order in which subjects are treated are excellent, and the matter of the work is ably and forcibly presented, evincing both experience and learning on the part of the author, and yet so plainly and concisely presented as will render it most acceptable as a text-book to the student of medicine. We are pleased to recommend it as such, and as presenting that happy medium between the extremity of detail on the one hand and the extremity of brevity on the other, both of which are objectionable to the student. We are pleased to notice also that where the metric system of measures is used the interpretation is given in the old table. Failure to do this in Dalton's and other late works has very much detracted from their convenience and usefulness to the student; and the more so to the older members of the profession with whom the learning of the metric system is like the learning of a new language, and is, therefore, very obnoxious. We commend the work as amongst the very best and latest that has been presented to the profession as a text book for students.

*The Southern Surgical and Gynecological Association.*—This Association includes all the Southern States, and its meetings will be held on the second Tuesday of September of each year at such places as may be selected by the Association.

The following are the officers elected :

Dr. W. D. Haggard, of Nashville, Tenn., President ; Drs. R. D. Webb and J. W. Sears, both of Birmingham, First and Second Vice-Presidents, respectively ; Dr. W. B. Davis, of Birmingham, Secretary ; and Dr. H. P. Cochrane, of Birmingham, Treasurer.

The Judicial Committee consists of the following :

Dr. John S. Cain, Nashville, Tenn.; Dr. Hunter McGuire, Richmond, Va.; Dr. J. M. Taylor, Corinth, Miss.; Dr. D. Saussure Ford, Augusta, Ga.; Dr. R. A. Kinloch, Charleston, S. C:

Dr. W. F. Hyer, of Holly Springs, Miss., was elected orator for the next annual session, which will be held in Birmingham.

Publication Committee.—Drs. W. E. B. Davis, R. D. Webb, and W. Locke Chew.

Committee of Arrangements.—Dr. J. S. Davis, Brice M. Hughes, J. C. Dozier, J. B. Luckie, and J. S. Gillespie.

SANDERS & SONS' EUCALYPTI EXTRACT (*Eucalyptol*).—Apply to Dr. Sanders, Dillon, Iowa, for gratis supplied reports on cures effected at the clinics of the Universities of Bonn and Griefswald.

*Visiting List.*—The Medical News' Visiting List. 1888. Philadelphia: Lee Brothers & Co.

This List, as prepared by the above publishing house, is as near perfection, it would seem, as could well be made. A great variety of Tables, Signs, Weights and Measures, Thermometric Scales, Calendars, Examination of Urine, Disinfectants, New Remedies, Incompatibilities, Therapeutic Table, Table of Doses, A Monthly and Daily Record of Entries, Obstetric Engagements, an Erasable Tablet for Memoranda, etc., etc. It is of convenient size to carry in the pocket, and is in all respects a most desirable book for the practitioner of medicine.

TYPHOID FEVER.—I was induced to employ Listerine by reading the article on scarlet fever by J. Lewis Smith, M. D., in Pepper's System of Medicine. It is the best restorative remedy I have yet found in diseases of the mucous surfaces.

I use it in my typhoid fever cases in teaspoonful doses every six hours. It promptly destroys all bad odors and taste in the mouth, and is very grateful to patient.

In a recent case of tonsillitis, the mouth and throat appearing a dirty, ragged slate color, accompanied by very bad odor, I requested patient to wash and gargle mouth and throat with Listerine, and the change was wonderful.—*Dr. Scott Russell, in Medical Brief.*

